# How stepping out helped us tune in: finding space and time to *think* as an early career researcher

**DOI:** 10.1038/s44323-024-00010-4

**Published:** 2024-10-01

**Authors:** Manuel Spitschan, Laura Kervezee, Renske Lok, Elise McGlashan, Raymond P. Najjar

**Affiliations:** 1 TUM School of Medicine & Health, Department of Health and Sport Sciences, Technical University of Munich, Munich, Germany; 2 TUM Institute for Advanced Study (TUM-IAS), Technical University of Munich, Garching, Germany; 3 Max Planck Institute for Biological Cybernetics, Max Planck Research Group Translational Sensory & Circadian Neuroscience, Tübingen, Germany; 4 Group of Circadian Medicine, Department of Cell and Chemical Biology, Leiden University Medical Center, Leiden, Netherlands; 5 Department of Psychiatry and Behavioral Sciences, Stanford University, Stanford, USA; 6 Melbourne School of Psychological Sciences, The University of Melbourne, Parkville, VIC Australia; 7 Department of Ophthalmology and Department of Biomedical Engineering, National University of Singapore, Singapore, Singapore; 8 Center for Innovation & Precision Eye Health, National University of Singapore, Singapore, Singapore; 9 Singapore Eye Research Institute, Singapore, Singapore

**Keywords:** Neuroscience, Diseases

## Abstract

The transition from postdoc to junior faculty is exciting and uniquely challenging. On one hand, it allows for increased creative freedom and the opportunity to grow into an independent scientist. On the other hand, it comes with increasing administrative responsibilities, feelings of isolation, and high pressure to perform. The result is an environment that can leave very limited time for creative thinking and reflection. Here, we describe how participating in a program that allowed us to step out of our routine and work together helped us become more independent—and regain time to think.

Over the past three years, we participated in a program that allowed us to meet at different locations across Europe for a week at a time, away from our home institutions. This experience made us realize how valuable it is to step away from the daily routine. Every year, the Network of European Institutes of Advanced Study (NETIAS) invites applications by groups of early-career researchers to spend work visits at a selection of these institutes. NETIAS is a consortium of university-adjacent institutes modeled after the original Institute for Advanced Study in Princeton, NJ, which, since 1930, has given space and time to scholars (including Albert Einstein, John von Neumann and Emmy Noether) to focus on research^1^. The NETIAS Constructive Advanced Thinking program (http://netias.science/project_constructive-advanced-thinking), aimed at developing networks of early-career researchers working on a common topic—through an interdisciplinary lens—provided this opportunity for us. We are a group of five early-career researchers based in five different countries (Australia, Netherlands, Germany, Singapore, and United States) on four continents (Australia, Europe, Asia, and America) from different backgrounds (psychology, biomedical science, neuroscience), brought together by an interest in the non-visual effects of light on physiology and behavior—and the desire to ‘zoom out’ and take stock of the research we do. We spent one week each at the Aarhus Institute of Advanced Study (Aarhus, Denmark), the Zukunftskolleg Konstanz (Konstanz, Germany), MAK’IT (Montpellier), and two weeks at the Wissenschaftskolleg zu Berlin (Berlin, Germany). At the beginning of the program (2021), we were all postdocs, and have since made or are making the transition to junior faculty.

Our time away allowed us to focus on areas that are important but are hard to fit in alongside our day-to-day research and teaching activities. Within our project group, we tackled a key problem in the field: The lack of harmonized reporting for light interventions. In our survey of the literature, we found a lack of standard quantities being reported in studies, leading to the unfortunate situation that studies cannot be directly compared in terms of the stimulus. To address this knowledge, we implemented a consensus process with 60 international experts, leading to the ENLIGHT Checklist^2^. The ENLIGHT Checklist provides a systematic structure for reporting light interventions, and has already been endorsed by a series of scientific organizations in our field (Society for Research in Biological Rhythms, Sleep Research Society, Society for Light Treatment and Biological Rhythms, Australasian Chronobiology Society, Center for Environmental Therapeutics, and the Circadian Mental Health Network), funders, and journals (including *npj Biological Timing and Sleep*).

We also formed valuable friendships and had the physical and mental space to reconnect with scientific creativity and allowed us *to think*^3^.

What contributed to our positive experience? Despite most of us having never met in person before our first meeting and our diverse backgrounds, our group shared a unified vision of what we wanted to accomplish. This made our group more cohesive, facilitated our discussions, and enhanced our productivity. At the base, personality characteristics also played a role. As self-described, easy-going, respectful individuals, we enjoyed chatting about science, personal experiences, and professional insights. Our common characteristics allowed us to quickly develop strong friendships. The environment was key: Our host institutions offered an ideal, protected, and neutral setting for our focused discussions, along with plenty of coffee to keep us going. Most importantly, each one of us was committed to making this successful. We dedicated time off from our day-to-day routines, ensured consistent communication even during the COVID-19 pandemic through Zoom, and above all, we understood the importance of our collective effort for our field and our career, and relied on one another to accomplish our group’s objectives.

The experience has been extremely valuable for our immediate career progression, and we have also gained new perspectives and insights that we take with us. One is the importance of having support from peers at the same or similar career stage. At the doctoral level, this may be a given, but it becomes less common later on. Having forged our connections, we will continue to share our professional challenges and provide mutual support and advice. Moreover, it taught us the importance of taking a break from our regular duties and dedicating time to think and reflect on our objectives and ambitions.

Moving forward, we will actively look for opportunities to clear our calendars, making space for protected time for creativity. Likewise, having reaped the benefits from this experience, we will encourage our trainees to pursue such opportunities as well, requiring, of course, to grant them the same flexibility that we were granted. Lastly, our experience highlighted the importance of selecting the right collaborators. Looking back, we realize how effortlessly we were on the same wavelength. Yet, this fit isn’t guaranteed, underlining how much good collaborative science is driven by interpersonal factors.

Being away from our home institutions—with their demands in research, teaching and administration—allowed us to recapture creativity, while developing a supportive peer network. It is not slowing down, but rather investing in future productivity and perspective. We hope that funding agencies, institutes, and mentors continue to create and sustain similar initiatives for postdocs (e.g. in the form of postdoctoral consortia^4^) as an investment in future generations of scientists—allowing them to reclaim the space and time to do something that often slips off the radar: *to think*.
